# An Interstitial 20q11.21 Microdeletion Causing Mild Intellectual Disability and Facial Dysmorphisms

**DOI:** 10.1155/2013/353028

**Published:** 2013-02-14

**Authors:** Ivan Y. Iourov, Svetlana G. Vorsanova, Oxana S. Kurinnaia, Yuri B. Yurov

**Affiliations:** ^1^Mental Health Research Center, Russian Academy of Medical Sciences, Moscow 119152, Russia; ^2^Institute of Pediatrics and Children Surgery, Ministry of Health of the Russian Federation, Moscow 125412, Russia; ^3^Moscow City University of Psychology and Education, Moscow 127051, Russia

## Abstract

We report a case of an interstitial chromosome 20q11.21 microdeletion in a 7-year-old male child presenting with mild intellectual disability and facial dysmorphisms. Array comparative genomic hybridization (CGH) has shown that the deletion resulted in the loss of 68 genes, among which 5 genes (*COX4I2*, *MYLK2*, *ASXL1*, *DNMT3B*, and *SNTA1*) are disease causing. The size of the deletion was estimated to span 2.6 Mb. Only three cases of deletions encompassing this chromosomal region have been reported. The phenotype of the index patient was found to resemble the mildest cases of Bohring-Opitz syndrome that is caused by *ASXL1* mutations. An *in silico* evaluation of the deleted genomic region has shown that benign genomic variations have never been observed to affect the *ASXL1* gene, in contrast to the other disease-causing genes. As a result, it was suggested that *ASXL1* loss is likely to be the main cause of the phenotypic manifestations. The present case report indicates that a loss of the disease-causing gene can produce a milder phenotype of a single gene condition.

## 1. Introduction

The application of array comparative genomic hybridization (CGH) in clinical cytogenetics has significantly increased the diagnostic yield [1, 2]. Moreover, studying genome variations in neurobehavioral diseases using array CGH has promoted the identification of new causative submicroscopic chromosome imbalances in the clinical population [2, 3]. As a result, array CGH molecular cytogenetic analysis has become almost indispensable in children suffering from intellectual disability and related neurobehavioral problems [1–3].

Performing a similar study in the Russian cohort of children with intellectual disability and congenital malformations (for details see [4]), we have identified an interstitial 20q11.21 microdeletion in a 7-year-old male child presenting with mild intellectual disability and facial dysmorphisms. According to the available literature, only three cases of chromosome 20 deletions encompassing the same chromosomal region (excluding somatic chromosome rearrangements associated with malignant pathology) have been reported and only two cases of interstitial deletions involving 20q11.21 were previously characterized by array CGH [5–7].

## 2. Case Presentation and Methods

### 2.1. Clinical Description

A 7-year-old male child was referred to molecular cytogenetic analysis, because of intellectual disability and facial dysmorphisms. He was born at 39 weeks of gestation to a 25-year-old mother and 28-year-old father. The couple is healthy and unrelated, having a history of a previous first trimester miscarriage. The pregnancy was reported to be complicated by hypertension during the third trimester. He was delivered vaginally. Neonatal measurements were as follows: birth weight was 2.9 kg (10th centile), and length was 49 cm (25th centile). At the age of one week, feeding problems (feeding intolerance and food refusal) were noted. Physical examination made at the age of  7 years showed microretrognathia, hypertelorism, upslanting palpebral fissures, prominent eyes, broad nasal bridge, low set ears, and low frontal hairline. Mild intellectual disability and speech delay were noticed.

### 2.2. Cytogenetic Analysis

Cytogenetic analysis was performed by GTG-banding according to standard procedures. Thirty metaphase plates were studied at a resolution higher than 550 bands. Detectable karyotype abnormalities were not found.

### 2.3. High-Resolution Metaphase CGH

**Figure 1:**
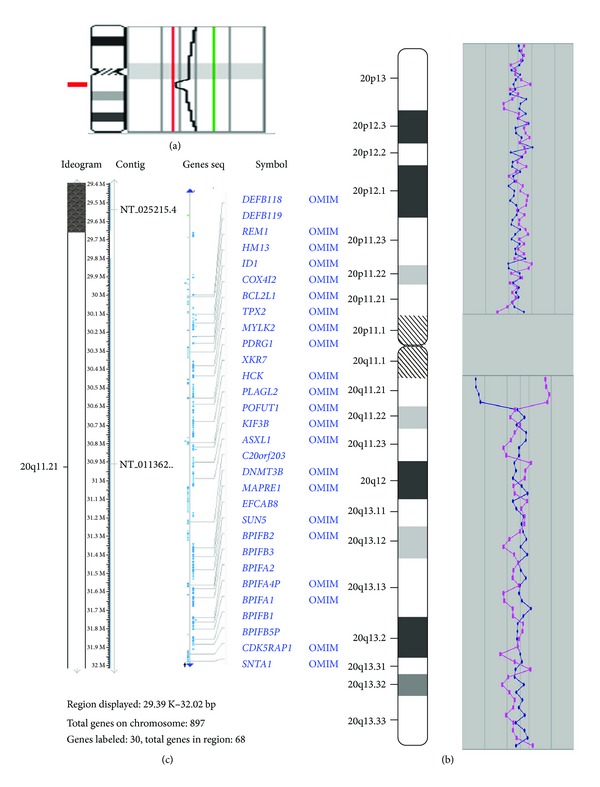
Molecular cytogenetic (CGH) findings in the index case: (a) high-resolution metaphase CGH demonstrating ish cgh dim(20)(q11.21q11.21); (b) array CGH demonstrating arr 20q11.21(29,392,835-32,017,043)x1 (two alternative arrays Cy3/Cy5 (pink line) and Cy5/Cy3 (blue line) were plotted on the graph); (c) and depiction of the deleted chromosomal region by NCBI Build 37.1/NCBI Map Viewer (http://www.ncbi.nlm.nih.gov/projects/mapview/map_search.cgi?taxid=9606).

High-resolution metaphase CGH was performed according to previously described protocols of DNA labeling, hybridization, and detection [8, 9]. The use of this technique has suggested the presence of a deletion in 20q11.2 (Figure 1(a)).

### 2.4. Array CGH

Array CGH was done using the customized human genomic microarrays (slightly modified Constitutional Chip 4.0) containing about 5000 human BAC/PAC clones (Human BAC Array-System, Perkin Elmer, USA) achieving a resolution of 0.3–1 Mb for the whole genome scan. Technical performance of array CGH (DNA labeling, hybridization, detection, and data analysis) was made according to previously described protocols [4, 10, 11] and to manufacturers' instructions. Array CGH has revealed an interstitial deletion in 20q11.21 spanning from 29,392,835 to 32,017,043 (confirmed by four BAC probes: RP5-1018D12, RP5-836N17, RP5-1085F17, and RP5-1125A11 in two reverse assays). The minimal deletion size was estimated to be about 2.6 Mb (Figure 1(b)).

### 2.5. *In Silico *Evaluation of the Deleted Chromosomal Region

**Figure 2:**
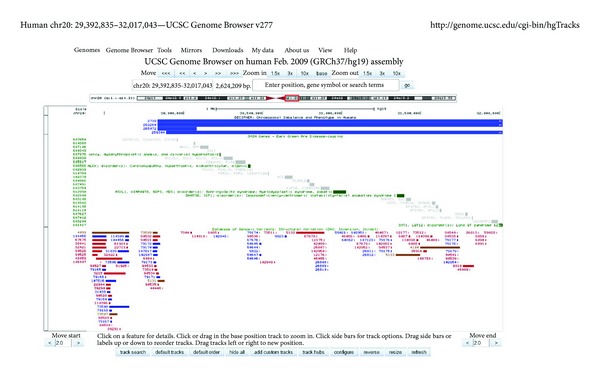
*In silico *evaluation of the deleted region by UCSC Genome Browser (GRCh37/hg19) (http://genome.ucsc.edu/), including data retrieved from DECIPHER (database of unbalanced chromosome aberrations http://decipher.sanger.ac.uk/), OMIM (http://www.omim.org/) (dark green OMIM genes are disease causing), and Database of Genomic Variants (http://dgvbeta.tcag.ca/dgv/app/home?ref=GRCh37/hg19) (blue bars correspond to a gain in size relative to the reference; red bars correspond to a loss in size relative to the reference; brown bars correspond to both losses and gains in size relative to the reference.).

To get further insights into understanding of the phenotypic outcome, we evaluated the deleted region by NCBI Build 37.1 (Figure 1(c)) and UCSC Genome Browser (Figure 2) as previously described [4]. The deletion resulted in the loss of 68 genes, among which 23 genes are listed in OMIM (http://www.omim.org/). Among the latter, five genes are disease causing:* COX4I2* (exocrine pancreatic insufficiency, dyserythropoietic anemia, and calvarial hyperostosis/OMIM: 612714), *MYLK2* (cardiomyopathy, hypertrophic, midventricular, and digenic/OMIM: 192600), *ASXL1* (Bohring-Opitz syndrome/OMIM: 605039 and myelodysplastic syndrome, somatic/OMIM: 614286), *DNMT3B* (immunodeficiency-centromeric instability-facial anomalies syndrome 1 or ICF syndrome/OMIM: 242860), and *SNTA1 *(long QT syndrome 12/OMIM: 612955). It is noteworthy, that no similar cases are indexed in DECIPHER (database of unbalanced chromosome aberrations http://decipher.sanger.ac.uk/) and benign genome variations (retrieved from Database of Genomic Variants http://dgvbeta.tcag.ca/dgv/app/home?ref=GRCh37/hg19) mapped to this genomic region involve *DNMT3B* (Figure 2).

## 3. Discussion

We present a case of an interstitial microdeletion (2.6 Mb) in 20q11.21 that has resulted in a loss of 68 genes. The chromosomal region was previously reported to be deleted in three cases (deletions of 6.5, 6.6, and 6.8 Mb within 20q11.2–q12) [5–7]. Generally, interstitial deletions of the long arm of chromosome 20 are rare. Apart from the previously mentioned three cases, another ten cases of intellectual disability (mental retardation) and congenital anomalies were associated with deletions within the long arm of human chromosome 20 (for more details see [7]). Clinically, cases of deletions in chromosome 20q11.2–q12 are similar to the present one. However, the index case exhibits a significantly milder phenotype compared to previous ones. For instance, intellectual disability and facial dysmorphisms were all more severe in cases demonstrating deletions of 20q11.2–q12. Interestingly, all the facial dysmorphisms and feeding problems are observed not only in cases of 20q11.2–q12 loss, but also in Bohring-Opitz syndrome. The latter condition is usually the result of mutations in *ASXL1 *[12]. To be more precise, addressing the study of Hoischen and colleagues [12], we have noticed that feeding problems, microretrognathia, hypertelorism, upslanting palpebral fissures, prominent eyes, broad nasal bridge, low set ears, and low frontal hairline are all observed in *ASXL1* mutation positive cases. On the other hand, patients with Bohring-Opitz syndrome usually exhibit much more severe phenotype as to the index case (i.e., severe (profound) intellectual disability; growth retardation; and craniofacial, ophthalmic, and neurological abnormalities). The phenotypic differences can be explained by a suggestion that *ASXL1* mutations cause loss of functions, whereas an allelic loss is likely to result in *ASXL1* dosage decrease that is probably less severe if the gene is not mutated.

Apparently, phenotypic manifestations of diseases caused by genomic variations within the remaining four genes were not observed in the present case. In this context, one can propose that an allelic loss of these genes can be benign and is likely to be observed in unaffected individuals. *In silico* evaluation of the deleted genomic region has shown that benign genomic variations have never been observed to affect the *ASXL1* gene, in contrast to the other disease-causing genes within this chromosomal region. Moreover, the loss of *DNMT3B*, mutations in which cause immunodeficiency-centromeric instability-facial anomalies syndrome, appears not to contribute to the present phenotype. On the other hand, benign variations encompassing this gene have been reported (Figure 2) allowing a suggestion that *DNMT3B* allelic losses can be benign in some cases. Taking into account the data acquired by *in silico* analysis, we have proposed that *ASXL1* loss is the main cause of phenotypic abnormities in this case. We have to add that the presence of an *ASXL1 *mutation is unlikely in the index case according to comparative analysis between Bohring-Opitz syndrome and 20q11.21 microdeletion phenotypes. Thus, the present case might be considered as an example of nonmutated *ASXL1 *loss, which is likely to be associated with milder phenotypes of Bohring-Opitz syndrome. Nonetheless, the loss of genes, which are not associated with a specific disease, can contribute to the phenotype, as well. Finally, somatic mosaicism, which reduces the consequences of genomic imbalances and is a relatively frequent occurrence among structural chromosome abnormalities [13], can be theoretically considered as a potential cause of mild phenotypic manifestations. In conclusion, the present case report indicates that a deletion can lead to a milder phenotype of a single gene condition and the loss of neighboring disease-causing genes due to the same deletion can be benign. To our knowledge, this is the first case of 20q11.21 microdeletion that is associated with such a mild phenotype.
